# Recent Modification Strategies of MoS_2_ for Enhanced Electrocatalytic Hydrogen Evolution

**DOI:** 10.3390/molecules25051136

**Published:** 2020-03-03

**Authors:** Chao Meng, Xiaodong Chen, Yuanfeng Gao, Qianqian Zhao, Deqiang Kong, Mengchang Lin, Xuemin Chen, Yuxia Li, Yue Zhou

**Affiliations:** 1 Key Laboratory for Robot and Intelligent Technology of Shandong Province, Shandong University of Science and Technology, Qingdao 266590, China; cxd19951119@163.com (X.C.); 15689976625@163.com (Y.G.); zqqhello@126.com (Q.Z.); k1305436964@163.com (D.K.); mengchanglin@sdust.edu.cn (M.L.); yuxiali2004@vip.163.com (Y.L.); 2 College of Science, Hebei University of Science and Technology, Shijiazhuang 050018, China; chxm0058@163.com

**Keywords:** molybdenum disulfide, modification strategies, hydrogen evolution, intrinsic activity, active sites

## Abstract

Molybdenum disulfide (MoS_2_) has been recognized as one of the most promising catalysts to replace Pt for hydrogen evolution reaction (HER) electrocatalysis because of the elemental abundance, excellent catalytic potential, and stability. However, its HER efficiency is still below that of Pt. Recent research advances have revealed that the modification of pristine MoS_2_ is a very effective approach to boost its HER performance, including improving the intrinsic activity of sites, increasing the number of edges, and enhancing the electrical conductivity. In this review, we focus on the recent progress on the modification strategies of MoS_2_ for enhanced electrocatalytic hydrogen evolution. Moreover, some urgent challenges in this field are also discussed to realize the large-scale application of the modified-MoS_2_ catalysts in industry.

## 1. Introduction

Hydrogen, with its high energy density (143 kJ/g) and pollution-free combustion product (water), has been considered the most potential new energy to replace fossil fuels in the 21st century [1,2]. Compared to conventional hydrogen production technology via steam methane reforming (i.e., CH_4_ + 2H_2_O → CO_2_ + 4H_2_), electrocatalytic hydrogen evolution from water splitting is more environmentally friendly [3,4,5]. At present, the most efficient hydrogen evolution reaction (HER) electrocatalyst reported is Pt, which can effectively lower the overpotential to near zero and consequently improve the efficiency of HER [6]. However, the high price and scarce reserve restrict its large-scale application. Therefore, it remains challenging to develop highly active HER catalysts with lower cost and higher abundance.

For the last decade and more, MoS_2_ has drawn wide attention for the electrocatalytic HER, mainly due to the earth abundance and excellent stability [2,7,8,9]. However, the HER catalytic efficiency of MoS_2_ is still far from that of Pt [10,11], so numerous efforts have been made on the modification of MoS_2_ to improve its HER performance. Except for enhancing the charge transport by defect engineering [12] and phase transition [13], the most popular method is to create more edges in MoS_2_, because its edges are more active than the inert basal plane for HER [14,15]. Ajayan et al. successfully adopted oxygen plasma and hydrogen annealing technologies to introduce abundant cracks and holes inside monolayer MoS_2_, respectively, which obviously increased the number of active edges and thus improved the total HER activity of the original MoS_2_ [16]. Another effective strategy for modifying MoS_2_ is to improve the intrinsic activity of the sites, including the optimization of the electronic structure of edge sites and the activation of the inherently inert basal plane [17,18]. Recently, Luo et al. reported the modification of pristine MoS_2_ by single-atom Pd doping [19]. The doped Pd atoms induced a distorted basal plane and, as a result, formed new electronic states to adjust the hydrogen adsorption behavior on the coordinated S atoms, which led to the enhancement in the intrinsic activity of pristine MoS_2_. Besides, Li et al. reported that the HER activity of monolayer MoS_2_ can be improved via the introduction of abundant S vacancies and strains, which tuned its band structure and reduced the free energy change for hydrogen adsorption (∆*G*_H*_) [20]. Apart from the above doping and vacancy engineering, optimized and enhanced properties can also be achieved by ion intercalation [21], surface functional group modification [22], and so on.

Given the rapid progress and new breakthroughs of MoS_2_ modification for enhanced hydrogen evolution, a detailed review is urgently needed to cover this emerging field and guide its further development. As far as we know, most of the published reviews have focused on the structure, synthesis, and electrochemical applications of MoS_2_ [3,13,23,24,25,26,27], with little detail on its modification methods to improve the HER performance. In this review, we summarize the recent modification strategies of MoS_2_ in detail for boosting its HER activity. Firstly, we introduce the structural characteristics of MoS_2_ and unfavorable factors that limit its catalytic efficiency. Secondly, the recently reported modification approaches of MoS_2_ are summarized and discussed from the following three aspects, including the improvement in the intrinsic activity of sites, the increase in edge sites, and the electrical conductivity enhancement. Finally, a brief discussion on some issues to be solved before using the modified MoS_2_ catalysts in industry is described.

## 2. Structural Characteristics of MoS_2_

**Figure 1:**
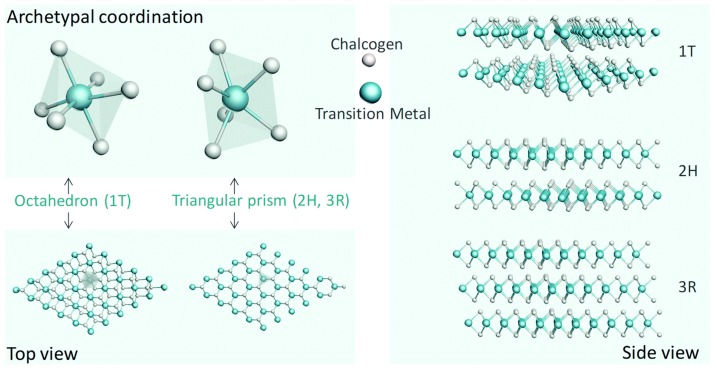
The typical 1T, 2H, and 3R structures of MoS_2_ (top and side view). Reproduced with permission from Ref. 24. Copyright © 2018, Royal Society of Chemistry.

As a typical lamellar hexagonal structure, MoS_2_ has separate S–Mo–S layers that interact with each other by weak van der Waals forces [28]. Generally, the Mo atoms in MoS_2_ are covalently bonded to six adjacent S atoms, and there are no dangling bonds between layers [29]. From the point of view of crystallography, the possible phase structures of MoS_2_ include 1T-, 2H-, and 3R- types. The stacking methods for both 2H- and 3R-types are the “A-B-A” method, and the Mo atoms occupy the center of triangular prisms [24]. The main structural difference between them is that the 2H-type has two layers per unit cell along the c-axis, whereas for the 3R-type, it possesses three layers per unit cell (Figure 1) [3]. Once heated, the 3R-type is easily converted to 2H, meaning that the 2H-type is more stable than the 3R-type. Unlike the stacking method of “A-B-A” in the 2H and 3R phases, the stacking method in the 1T phase is “A-B-C” and the Mo atoms are at the center of the octahedral structure (Figure 1) [24]. The metastable metallic 1T phase can be obtained via chemical Li-intercalation and exfoliation of the 2H phase with the semiconducting property, and the phase transition from 2H to 1T shows a positive effect for enhancing HER performance [30].

A number of studies have reported that the catalytic sites in MoS_2_ for HER electrocatalysis are mainly from the active Mo edges rather than the inert basal plane [31,32]. However, notably, the ∆G_H*_ associated with intrinsic activity of the 2H-MoS_2_ edge site is still a little far from the optimal value of zero [9,11,19]. Therefore, we think that there are three main strategies for the modification of pristine MoS_2_ to improve its HER performance: (1) Improving the intrinsic activity of sites by appropriately tuning the electronic structure of the edge, or activating the inherently inert basal plane to optimize the bond strength of the adsorbed H; (2) increasing the number of edge active sites via creating cracks or holes inside the MoS_2_ layers; (3) improving the conductivity by introducing new electronic states near the Fermi level and narrowing the band gap of the original MoS_2_. Along these strategies, various approaches have been developed to modify MoS_2_ for the HER performance enhancement, and the details will be described below.

## 3. Modification Strategies for Boosting the HER Activity of MoS_2_

### 3.1. Improving the Intrinsic Activity of Sites

#### 3.1.1. Metal Cation Doping

**Figure 2:**
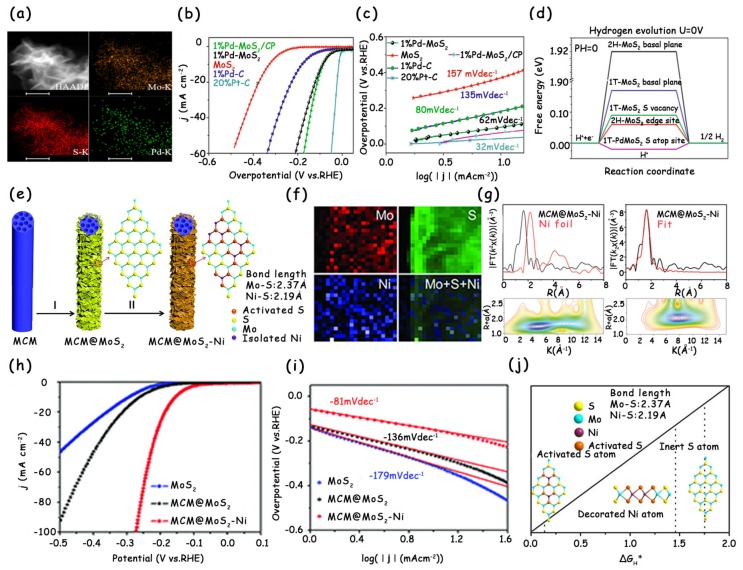
(**a**) High-angle annular dark-field (HAADF) and elemental mappings for S, Mo, and Pd of Pd–MoS_2_. (**b**) Linear sweep voltammetry (LSV) polarization curves and (**c**) corresponding Tafel slopes of MoS_2_, 1% Pd–MoS_2_, 1% Pd–MoS_2_/CP, 1% Pd–C, and 20% Pt–C. (**d**) Free energy versus the reaction coordinates of different active sites. (**e**) Schematic illustration of the synthetic process for MCM@MoS_2_–Ni: I) Growth of MoS_2_ nanosheets, II) surface decoration of isolated Ni atoms. (**f**) EELS mapping image of MCM@MoS_2_–Ni. (**g**) Characterization of the single-atom structure of Ni in MCM@MoS_2_–Ni. (**h**) LSV curves and (**i**) corresponding Tafel plots of MoS_2_, MCM@MoS_2_, and MCM@MoS_2_–Ni in 0.5 M H_2_SO_4_. (**j**) Theoretical calculations for the effects of Ni decoration on the hydrogen evolution reaction (HER) activity of MoS_2_. (**a**–**d**) have been reproduced with permission from Ref. 19. Copyright © 2018, Nature Publishing Group. (**e**–**j**) have been reproduced with permission from Ref. 18. Copyright © 2018, Wiley-VCH Verlag GmbH & Co. KGaA, Weinheim.

In order to activate the basal plane of MoS_2_, one of the most effective approaches is the doping of metal cations into MoS_2_ to form heterogeneous catalysts, including noble metal-doping and non-noble metal doping [18,19,33]. After metal cation doping, due to the difference in the bond lengths and angles of Mo–S and X–S (like Pd, Ru, and Ni) bonds, the doped cations will produce a distorted configuration over the basal plane and form new electronic states to tune the adsorption behavior of H atoms on the coordinated S atoms. The optimized H adsorption strength eventually leads to the improvement in the intrinsic activity of original MoS_2_ [18,19,33]. For example, Luo et al. successfully modified MoS_2_ by single-atom Pd doping (Figure 2a), and the resultant 1% Pd–MoS_2_/CP exhibited an excellent electrocatalytic HER performance (an overpotential at 10 mA cm^−2^ of only 89 mV and a Tafel slope of 80 mV dec^−1^) (Figure 2b,c) [19]. By density functional theory (DFT) calculation analysis, the authors found that compared to the 1T-MoS_2_ basal plane, the energy barriers for adsorption and desorption after Pd-single-atom doping were obviously optimized, and the total energy ∆*G*_H*_ was closer to zero (Figure 2d).

Although the doping of precious metal Pd can significantly enhance the intrinsic activity of MoS_2_, its high cost limits the large-scale application. Recently, Lou et al. prepared Ni-modified MoS_2_ for enhanced electrocatalytic hydrogen evolution (Figure 2e) [18]. The electron energy loss spectroscopy (EELS) mapping (Figure 2f), extended X-ray absorption fine structure (EXAFS) spectra, and wavelet transform (WT) for the *k*^3^-weighted EXAFS signals together confirm that the existence of Ni in MoS_2_ nanosheets is in the form of single atoms (Figure 2g). The polarization curve and Tafel slope show that the HER performance after doping is much higher than that of undoped MoS_2_, and the improved performance is largely attributed to the activated basal planes of MoS_2_ (Figure 2h,i). The authors further conducted DFT calculations to investigate the changes in the Δ*G*_H*_ after the single-atom Ni modification (Figure 2j). For the inert basal plane of MoS_2_, Δ*G*_H*_ was calculated to be 1.75 eV, whereas the S-atop sites activated by the decorated Ni atoms resulted in a much-reduced Δ*G*_H*_ of 0.15 eV, which approaches that of edge S sites (0.08 eV). Besides, the Δ*G*_H*_ of the isolated Ni sites was determined to be 1.43 eV, indicating its little contribution to the HER activity. Doping MoS_2_ with precious or non-precious metals can modify the electronic structure of inert S sites on the basal plane and optimize their adsorption of H atoms, thereby resulting in enhanced intrinsic HER activity. However, the strategy of modifying MoS_2_ via cation doping, especially single-atom doping, usually involves time-consuming and tedious synthesis steps, and, as a result, it may not be suitable to prepare large quantities of catalysts efficiently.

#### 3.1.2. Anion Doping

**Figure 3:**
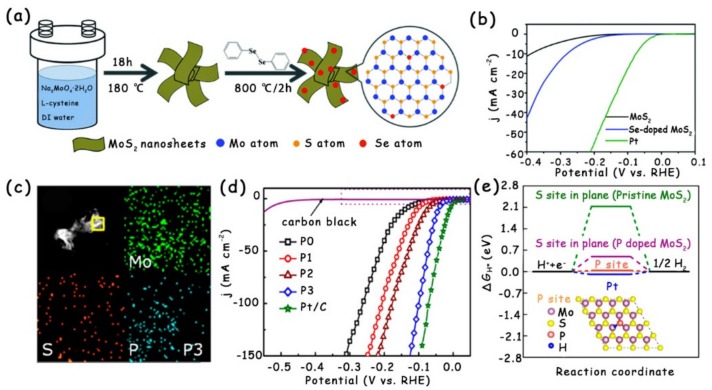
(**a**) Schematic illustration of synthesis procedure for the Se-doped MoS_2_ nanosheets. (**b**) Polarization curves of MoS_2_, Se-doped MoS_2_, and Pt electrode at the scan rate of 10 mV s^−1^ in 0.5 M H_2_SO_4_. (**c**) The element mapping result of P-doped MoS_2_ nanosheets. (P3 represents that the addition of NH_4_H_2_PO_4_ is 0.4 g.) (**d**) LSV curves of pure and P-doped MoS_2_ nanosheets in 0.5 M H_2_SO_4_. The bare carbon black is also chosen as the reference sample. (**e**) HER free-energy diagram for P site and S sites in the basal plane of pristine and P-doped MoS_2_. Insets show the P-doped MoS_2_ basal plane with an H atom adsorbed on the most active P site. (**a**,**b**) have been reproduced with permission from Ref. 34. Copyright © 2015, Royal Society of Chemistry. (**c**–**e**) have been reproduced with permission from Ref. 36. Copyright © 2017, American Chemical Society.

Once the anions are doped into MoS_2_ to replace the S atoms, more disorderly and defective lattices will appear in the new structure due to the size difference between S atoms and doped anions (like O, Se, and P) [34,35,36]. The distorted crystal structure naturally gives rise to the regulated electronic structure of active sites, which ultimately enhances their intrinsic activity [36]. Liu et al. doped Se into MoS_2_ via high-temperature annealing with diphenyl diselenide (DDS) as the Se source (Figure 3a) [34]. In contrast to the weak HER performance of pristine MoS_2_, the Se-doped MoS_2_ showed a markedly better activity with a low onset overpotential of about 140 mV. Especially at the overpotential of 400 mV, the Se-doped MoS_2_ presented an extremely high cathodic current density of 42.7 mA cm^−2^, which is approximately four times that in the pristine MoS_2_ (11.2 mA cm^−2^) (Figure 3b). The authors attribute the HER performance enhancement to the improved active edge sites and electrical conductivity.

Moreover, Xue et al. successfully doped P into the original MoS_2_ and adjusted the P doping concentration (Figure 3c) [36]. After the MoS_2_ was doped with P atoms, both the onset overpotential and overpotential decreased gradually. Notably, the P-doped MoS_2_ with the most suitable doping concentration displayed the least onset overpotential of 15 mV and overpotential at 10 mA cm^−2^ of ~43 mV, while the pure MoS_2_ nanosheets only exhibited an onset overpotential of 88 mV and overpotential at 10 mA cm^−2^ of 140 mV, indicating that the doping of P atoms can usefully enhance the HER activity of MoS_2_ nanosheets (Figure 3d). By combining calculations with experiments, the authors demonstrate that P dopants serve as the new active sites in the basal plane of MoS_2_ with the ∆*G*_H*_ of 0.04 eV, which is comparable to that of Pt (Figure 3e). Very recently, Zhou et al. reported the incorporation of oxygen into the MoS_2_/Mo mesh (MoS_2_/MM) by a facile electrochemical anodic activation process [35]. The doping of O atoms produced more active sites in MoS_2_ with tuned ∆*G*_H*_, thus possessing much a better HER activity than that of the initial MoS_2_/MM. This electrochemical activation method has also been successfully employed to modify MoSe_2_ and MoP.

#### 3.1.3. Introducing S Vacancies

**Figure 4:**
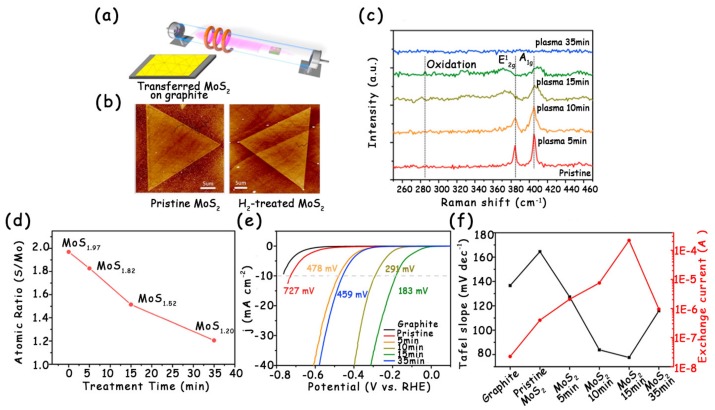
(**a**) Schematic illustration of the experimental setup for the surface treatment of MoS_2_ monolayers by inductively coupled H_2_-plasma. (**b**) AFM height images of a single crystalline MoS_2_ monolayer flake before and after hydrogen plasma treatment. (**c**) Raman spectra of the MoS_2_/graphite before and after H_2_-plasma treatment for varied time periods. (**d**) The atomic ratio of S/Mo for the monolayer MoS_2_ before and after plasma treatment. (**e**) The polarization curves of MoS_2_ with various hydrogen-plasma treatment periods at the scan rate 5 mV s^−1^ in 0.5 M H_2_SO_4_ solutions. (**f**) The corresponding Tafel slopes and exchange currents extracted from (**e**). Reproduced with permission from Ref. 14. Copyright © 2016, Elsevier Inc.

Creating S vacancies in the basal plane is crucial for improving the HER performance of MoS_2_. The introduction of S vacancies can activate the intrinsically inert basal planes and act as additional catalytic sites, eventually enhancing the HER activity of MoS_2_ [8,14,37,38]. Li’s group used the hydrogen plasma to introduce S vacancies into the basal plane of monolayer MoS_2_ (Figure 4a), and the concentration of S vacancies can be accurately adjusted by just changing the plasma treatment time (Figure 4d) [14]. As shown in Figure 4b, the geometrical structure and shape of MoS_2_ are well preserved after plasma treatment. However, the crystal symmetry of the plasma-treated MoS_2_ is destroyed (Figure 4c), indicating the presence of a large number of S vacancies. By comparing the linear sweep voltammetry (LSV) curves of pristine MoS_2_ and MoS_2_ treated with different times, the authors found that the MoS_2_ treated with 15 min plasma had the best HER performance (Figure 4f) and its overpotential at 10 mA cm^−2^ (183 mV) was much lower than that of pristine MoS_2_ (727 mV) (Figure 4e).

**Figure 5:**
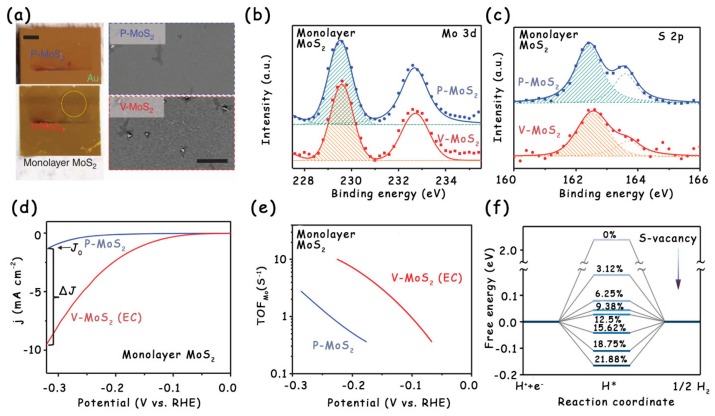
(**a**) Optical (left side; scale bar: 2 mm) and scanning electron microscopy (SEM) images (right side; scale bar: 20 mm) of monolayer MoS_2_ film before (P–MoS_2_, upper panel) and after (V–MoS_2_, lower panel) desulfurization. (**b**) X-ray photoelectron spectroscopy (XPS) Mo 3d and (**c**) S 2p spectra of P–MoS_2_ and V–MoS_2_. (**d**) LSV curves of monolayer MoS_2_ before and after desulfurization. (**e**) Turnover frequency (TOF) per surface Mo atom (TOF_Mo_) as a function of applied potential for P–MoS_2_ and V–MoS_2_. (**f**) Free energy diagram for the HER on S-vacancy sites with different vacancy concentrations. Reproduced with permission from Ref. 17. Copyright © 2017, Nature Publishing Group.

Although the plasma treatment is very efficient for the introduction of S vacancies into the basal plane of MoS_2_, this technology usually requires high-pressure synthesis conditions and is, therefore, not energy-saving enough [37,39]. Tsai et al. introduced a scalable route to generate S vacancies on the MoS_2_ basal plane using electrochemical desulfurization (Figure 5a–c) [17]. By changing the applied desulfurization potential, the content of S vacancies and the resulting HER activity can be easily varied. The experiment and calculation results showed that the optimal hydrogen adsorption free energy corresponds to an S-vacancy concentration between 12.5% and 15.62%, which results in a high per-site turnover frequency (TOF) (Figure 5e,f). However, it is worth noting that the desulfurized sample with vacancies (V–MoS_2_) had a limited total activity (Figure 5d), which must be optimized before replacing Pt-based catalysts for practical applications.

**Figure 6:**
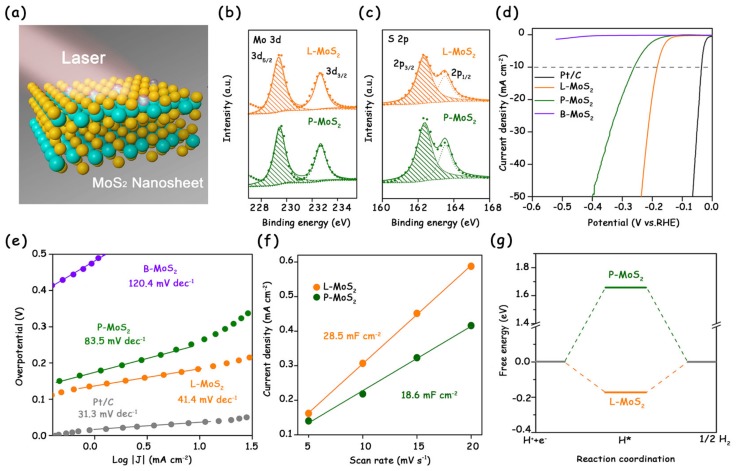
(**a**) Schematic illustration of MoS_2_ nanosheets treated by laser ablation in liquid (LAL). (**b**) XPS Mo 3d and (**c**) S 2p spectra of L-MoS_2_ and P-MoS_2_. (**d**) LSV curves of commercial Pt/C, L-MoS_2_, P-MoS_2_, and B-MoS_2_ recorded in 0.5 M H_2_SO_4_ with *iR*-correction. (**e**) Corresponding Tafel plots of MoS_2_ catalysts and Pt/C. (**f**) Linear fitting of the capacitive current density versus scan rate for L-MoS_2_ and P-MoS_2_. (**g**) Calculated free energy diagram of the HER on P-MoS_2_ and L-MoS_2_. Reproduced with permission from Ref. 7. Copyright © 2019, American Chemical Society.

Recently, our group first adopted the laser ablation in liquid (LAL) technique to introduce plentiful S vacancies on the basal plane of 2H-MoS_2_ nanosheets under ambient conditions (Figure 6a) [7]. The XPS spectra showed that the peak area ratio of S/Mo for the laser-treated MoS_2_ was smaller than that for pristine MoS_2_, indicating a S-vacancy concentration of about 8% in the laser-treated MoS_2_ (Figure 6b,c). Eventually, the laser-treated sample exhibited a low overpotential of 178 mV to reach 10 mA cm^−2^ and a Tafel slope of 41.4 mV dec^−1^, both of which far exceed those of pristine MoS_2_ (Figure 6d,e). We attribute the excellent HER performance to the additional active sites on the basal plane and the optimal ∆*G*_H*_ (Figure 6f,g).

#### 3.1.4. Strain Effect

**Figure 7:**
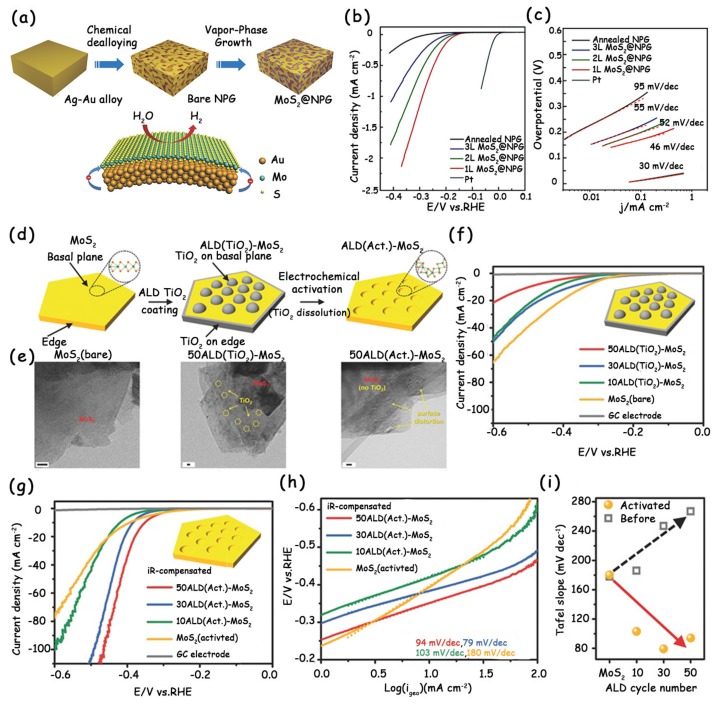
(**a**) Schematic diagram of the fabrication process of monolayer MoS_2_ on nanoporous gold (MoS_2_@NPG) hybrid materials by a nanoporous metal-based chemical vapor deposition (CVD) approach and schematic HER electrocatalysis. (**b**) Polarization curves and (**c**) the corresponding Tafel slopes of MoS_2_@NPG, annealed NPG, and Pt. (**d**) Schematic illustration of the atomic layer deposition (ALD) TiO_2_ coating on pristine MoS_2_ and subsequent electrochemical activation. (**e**) High-resolution transmission electron microscope (HRTEM) images of pristine MoS_2_ (bare), 50ALD(TiO_2_)-MoS_2_, and 50ALD(Act.)-MoS_2_. (**f**) LSV curves of pristine MoS_2_ and ALD(TiO_2_)-MoS_2_ samples with different cycles in a 0.5 M H_2_SO_4_ electrolyte. Note that the glassy carbon (GC) electrode is also tested as a reference. (**g**) *iR*-compensated LSV curves of pristine MoS_2_ (activated) and ALD(Act.)-MoS_2_ samples with different cycles, and (**h**) corresponding Tafel plots derived from (**g**). (**i**) Dependence of the Tafel slope and ALD cycle number before (square) and after (sphere) electrochemical activation. (**a**–**c**) have been reproduced with permission from Ref. 40. Copyright © 2014, Wiley-VCH Verlag GmbH & Co. KGaA, Weinheim. (**d**–**i**) have been reproduced with permission from Ref. 41. Copyright © 2017, Wiley-VCH Verlag GmbH & Co. KGaA, Weinheim.

Due to the atomic thickness and high elasticity, MoS_2_ can achieve lattice deformation through introducing strains. These strains produce highly localized surface distortions, which activate the inherently inert basal plane of MoS_2_ and thus benefits the hydrogen-binding. Ultimately, the modified-MoS_2_ samples can realize dramatically enhanced HER kinetics and activity [20,40]. Chen et al. reported that the direct growth of MoS_2_ on a curved surface of a nanoporous gold (NPG) can introduce out-of-plane strains into the monolayer MoS_2_ and cause a continuous change in bond angles (Figure 7a) [40]. The novel monolayer MoS_2_@NPG electrode shows excellent HER performance with a low onset potential of –118 mV and Tafel slope of 46 mV dec^−1^ (Figure 7b,c). However, notably, the NPG substrate used in this work is so expensive that it is difficult to achieve large-scale practical applications.

Recently, Kim’s group combined the atomic layer deposition (ALD) and electrochemical etching techniques, which are commonly used in the industry, to activate the inert basal planes of commercial bulk MoS_2_ [41]. In detail, they first deposited titanium dioxide (TiO_2_) islands on the MoS_2_ basal planes via ALD, which were then leached out using the in situ electrochemical activation method to form strains (Figure 7d). The electrocatalytic HER activities of these catalysts were tested using a three-electrode cell system in an aqueous 0.5 M H_2_SO_4_ solution (Figure 7f,g). As shown in the LSV curves (Figure 7g) and Tafel slopes (Figure 7h,i), the more cycles of ALD coating, the higher the catalytic performance of the activated MoS_2_. The authors attributed the reason to the more local surface distortions created on the MoS_2_ basal planes (Figure 7e).

#### 3.1.5. Forming Surface Functional Groups

**Figure 8:**
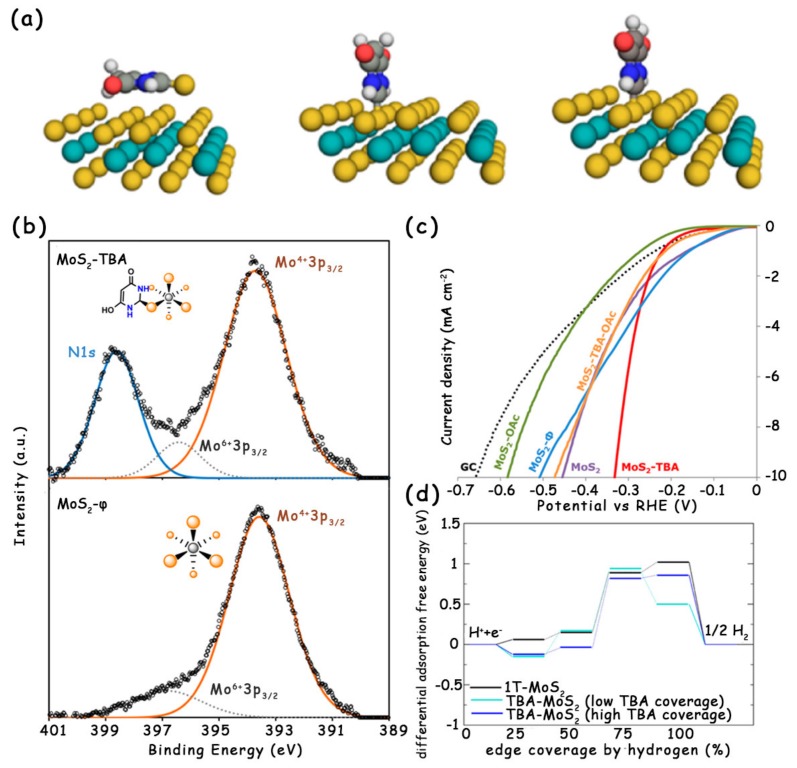
(**a**) Relaxed geometries of physisorbed (left) and two different covalently bound thiobarbituric acid (TBA) tautomers (middle and right). (**b**) XPS spectra of TBA-functionalized and unmodified MoS_2_. (**c**) LSV curves of MoS_2_ materials on GC at pH = 0. (**d**) Differential adsorption free energy of hydrogen on the edge of 1T-MoS_2_ and MoS_2_-TBA at 6% (low density) and 25% (high density, similar to a value determined by XPS) of TBA molecules with respect to surface S atoms. Reproduced with permission from Ref. 22. Copyright © 2017, American Chemical Society.

Modifying the MoS_2_ with surface functional groups can give rise to the charge transfer between them, which changes the electronic structure and hydrogen adsorption behavior of MoS_2_, thereby influencing its intrinsic HER activity [22,42]. For example, Pumera et al. studied the effect of the modification of different surface functional groups on the catalytic activity of MoS_2_ [22]. Among the numerous functional groups, the C–S covalent bond formed between MoS_2_ and thiobarbituric acid (TBA) was the only one that could enhance the HER activity of pristine MoS_2_ (Figure 8a–c). The authors attribute the reasons to the inherent 1T-phase metallic conductivity, weak surface basicity, and better wettability. Furthermore, they also analyzed the enhanced HER activity of MoS_2_-TBA with respect to the parent MoS_2_ from theoretical calculations. The calculation result showed a clear trend of improved catalytic activity at both low and high coverages of TBA. When the TBA coverage reaches 50%, the MoS_2_-TBA will become the most effective catalyst for electrocatalytic HER (Figure 8d), because the hydrogen atoms at the edge tend to avoid each other. Notably, the modification strategy of MoS_2_ by forming surface functional groups usually faces some drawbacks, including that (1) most of the surface functional groups the authors tried inhibit the HER activity of pristine MoS_2_; (2) the modification process takes a long time of more than 48 h; (3) as mentioned by the authors, it is difficult to achieve a balance between a high apparent yield of catalysts and high TBA content, because a less rigorous cleaning procedure results in a higher yield, but the TBA content will decrease with successive washes.

#### 3.1.6. H to 1T Phase Transformation

**Figure 9:**
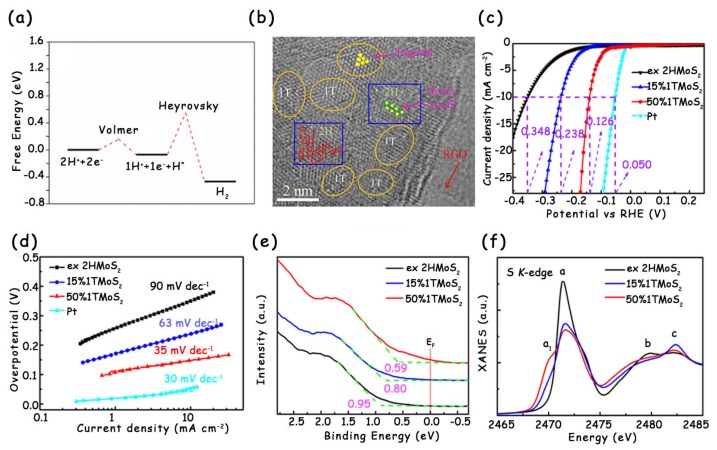
(**a**) Free energy diagram for the Volmer–Heyrovsky route on 1T-MoS_2_ at the electrode potential of –0.22 V vs. NHE (~25% surface H coverage). (**b**) HRTEM image of the 1T-2H MoS_2_ with a 1T content of 50%. (**c**) LSV curves and (**d**) corresponding Tafel slopes of ex-2H MoS_2_, 15% 1T MoS_2_, 50% 1T MoS_2_, and Pt. (**e**) Ultraviolet photoelectron spectra (UPS) and (**f**) S K-edge X-ray absorption near-edge structure (XANES) spectra of ex-2H MoS_2_, 15% 1T MoS_2_, and 50% 1T MoS_2_ nanosheets. (**a**) has been reproduced with permission from Ref. 46. Copyright © 2016, American Chemical Society. (**b**–**f**) have been reproduced with permission from Ref. 50. Copyright © 2017, American Chemical Society.

It is well-known that the rate-determining step for 2H-MoS_2_ to catalyze HER is the Volmer reaction [43,44,45]. Once the phase is transformed from 2H to 1T, the rate-determining step of MoS_2_ becomes the Heyrovsky reaction (Figure 9a) [46,47,48]. The phase transformation benefits improving the electrochemical reaction kinetics and optimizing the hydrogen adsorption behavior, so the HER activity of MoS_2_ is greatly enhanced [29,30,49]. Wei’s group reported a template-assisted strategy to modulate the 1T phase in 2H-MoS_2_ by reduced graphene oxide (RGO) (Figure 9b) [50]. RGO as the template can donate electrons to MoS_2_, which promote its phase transition. In comparison to pristine 2H-MoS_2_, the MoS_2_ modified with 50% metallic 1T phase exhibits obviously improved HER performance with a low overpotential at 10 mA cm^−2^ of 126 mV and an excellent Tafel slope of 35 mV dec^−1^ (Figure 9c,d). The electronic structure and electrochemical characterization reveal the significant electron density distribution near the Fermi level of 1T-2H MoS_2_ (Figure 9e,f), which accelerates the formation of H* intermediates and decreases the activation barrier of HER.

#### 3.1.7. Ion Intercalation

**Figure 10:**
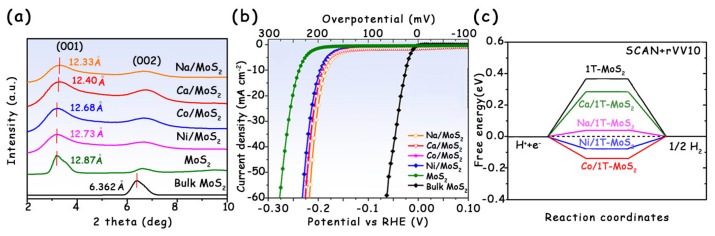
(**a**) Comparison of (001) and (002) X-ray diffraction (XRD) reflections of bulk 2H-MoS_2_, 1T-MoS_2_, and metal cation intercalated 1T-MoS_2_. (**b**) Polarization plots of current density (j) versus V after *iR* correction, showing enhanced catalytic activity of metal cation intercalated 1T-MoS_2_. (**c**) Free energies of hydrogen atom adsorption on the basal plane of pristine 1T-MoS_2_, as well as the intercalated 1T-MoS_2_. Reproduced with permission from Ref. 21. Copyright © 2017, American Chemical Society.

The intercalation of either a sp or *d*-band metal cation into the interlayer region of MoS_2_ can improve its efficiency for the HER [21,49]. On the one hand, the intercalated metal cations change the ∆*G*_H*_ [51]; on the other hand, a reduced charge on the sheets upon cation intercalation may allow more catalytic sites to be accessible for the proton reduction cycle, resulting in the increased electrochemical active surface area (ECSA) [52,53]. These two aspects together promote the electrocatalytic hydrogen evolution. Recently, Daniel et al. adopted a solution-based technique to intercalate different metal cations, including Na^+^, Ca^2+^, Ni^2+^, and Co^2+^, into 1T-MoS_2_ (Figure 10a), and tested their HER activity [21]. In acidic media, the onset potential of 1T-MoS_2_ with intercalated ions was about 60 mV lower than that of pristine 1T-MoS_2_ (Figure 10b). By combining with DFT calculations, the authors found a lowering in the Δ*G*_H*_ on these intercalated structures relative to intercalant-free 1T-MoS_2_. Especially for the 1T-MoS_2_ with Na^+^ intercalation, its Δ*G*_H*_ result was the closest to zero, indicating the excellent activity for the HER (Figure 10c).

### 3.2. Increasing the Number of Edge Active Sites

For typical MoS_2_, its edges are major active sites for HER electrocatalysis [15,54,55]. However, the edge only occupies a small portion of the surface area due to the layer structure of MoS_2_, thus leading to limited active sites and a relatively low catalytic activity [7,27,29]. In order to modify MoS_2_ via increasing the number of edge sites, defect engineering has been widely used like creating cracks or holes inside the MoS_2_ layers [16].

#### 3.2.1. Creating Cracks by High-Power Plasma

**Figure 11:**
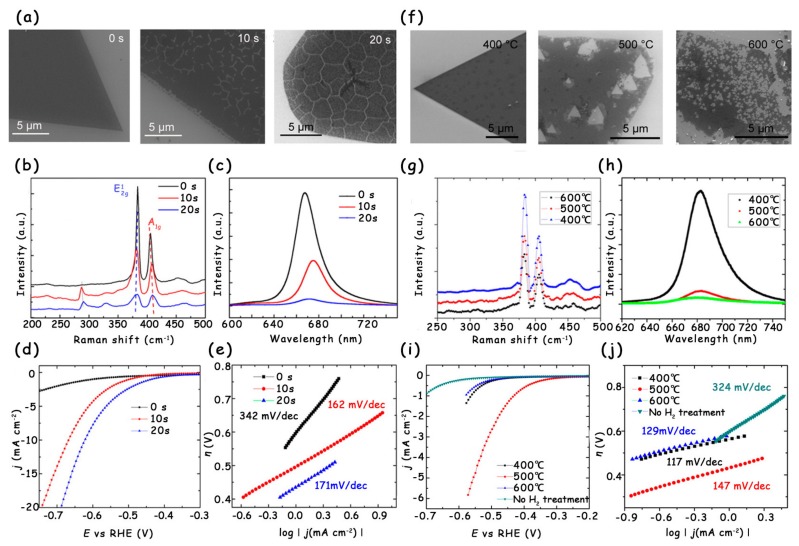
(**a**) SEM images, (**b**) Raman spectra, and (**c**) PL spectra of monolayer MoS_2_ with 0, 10, and 20 s oxygen plasma exposure. (**d**) LSV curves and (**e**) corresponding Tafel slopes of monolayer MoS_2_ with 0, 10, and 20 s oxygen plasma exposure. (**f**) SEM images, (**g**) Raman, and (**h**) PL spectra of monolayer MoS_2_ after H_2_ annealing at 400, 500, and 600 °C. (**i**) LSV curves of monolayer MoS_2_ with no H_2_ treatment and H_2_ annealing at 400, 500, and 600 °C. (**j**) The corresponding Tafel slopes derived from (**i**). Reproduced with permission from Ref. 16. Copyright © 2016, American Chemical Society.

By creating cracks inside pristine MoS_2_, more edge sites can be exposed to significantly improve the HER activity. Recently, Ajayan et al. reported the oxygen plasma technology to introduce abundant cracks into the monolayer MoS_2_ [16]. With the extension of plasma exposure time, both the width and density of the cracks increase (Figure 11a). However, once the exposure time is over 30 s, the high-power plasma easily causes excessive damage to the original morphology [12,37,38], which makes it difficult to transfer the MoS_2_ sample for further electrochemical measurement. The above result was further confirmed by Raman and photoluminescence (PL) spectra (Figure 11b,c). Eventually, the authors compared the LSV curves and Tafel slopes of monolayer MoS_2_ with 0, 10, and 20 s oxygen plasma exposure. As shown in Figure 11d,e, the MoS_2_ sample with 20 s exposure has the lowest overpotential and Tafel slope, which are attributed to the maximum number of edge active sites.

#### 3.2.2. Forming Holes via Hydrogen Annealing

Another effective approach for increasing edge active sites is the introduction of holes into MoS_2_ by hydrogen annealing, and the number of holes can be readily adjusted by changing the annealing temperature [16]. When the annealing temperature is set to 400 °C, no obvious morphology change is observed on the single-layer MoS_2_. Once the annealing temperature increases to 500 °C, the MoS_2_ begins to be etched and some small triangular holes appear. Nevertheless, when the annealing temperature rises to 600 °C, most of the MoS_2_ is decomposed because of the formation of high-density triangular holes (Figure 11f). The Raman and PL spectra clearly show the reduction in peak intensity after hydrogen annealing treatment, indicating the increase in defects and edge active sites in monolayer MoS_2_ (Figure 11g,h). To establish the relationship between the HER performance and the number of holes, the authors tested the LSV curves of MoS_2_ under different annealing temperatures. With the increase in the number of holes, the HER activity first improved and then decreased. The MoS_2_ annealed under 500 °C possessed the lowest onset overpotential (~300 mV) and largest current density, both of which are far better than those of the original MoS_2_ (Figure 11i,j). The reason the performance of MoS_2_ under 600 °C is worse than that of MoS_2_ under 500 °C is mainly ascribed to the fact that the left MoS_2_ with too many defects is not stable.

### 3.3. Improving the Electrical Conductivity

The original MoS_2_ with the 2H phase has the semiconductor characteristic with a band gap of 1.9 eV and weak charge transfer capability (a carrier mobility of 0.1–10 cm^2^ V^−1^ s^−1^) [56,57,58], which greatly restrict its HER performance. Recent studies have shown that the modification methods, such as phase transition, doping, and vacancy defect engineering, can introduce new electronic states near the Fermi level, which narrow the band gap of original MoS_2_ [33,59]. Meanwhile, the charge-transfer rate of the modified pristine MoS_2_ is also increased, meaning the improvement in the electrical conductivity.

#### 3.3.1. Synergistic Effect of Phase Transition and Doping

**Figure 12:**
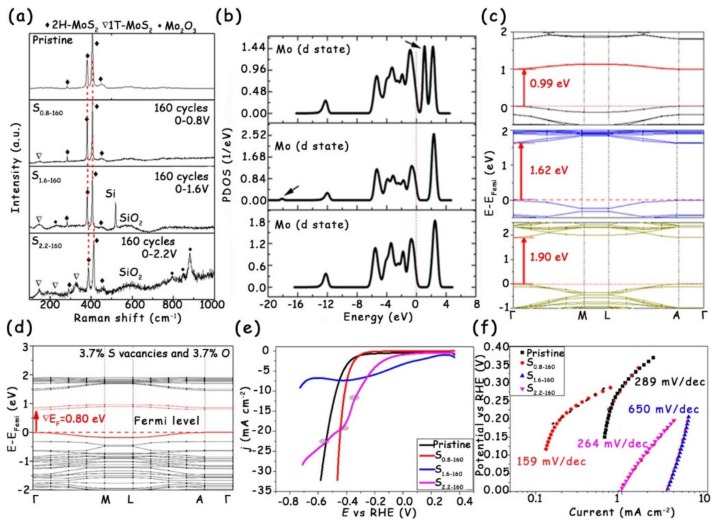
(**a**) Raman spectra of the multilayer 2H-MoS_2_ nanosheets treated under a varied scanning potential range. (**b**) Partial density of states (PDOS) of Mo-d and (**c**) calculated band structures for (3 × 3 × 1) 2H-MoS_2_ containing 3.7% S vacancies or 3.7% O atoms, and the pristine one (from top to bottom). (**d**) Band structures of (3 × 3 × 1) 2H-MoS_2_ with 3.7% O atoms and 3.7% S vacancies. (**e**) Polarization curves and (**f**) corresponding Tafel plots of the MoS_2_ nanosheets after electrochemical treatments with CVs at different scan potential ranges. Reproduced with permission from Ref. 59. Copyright © 2018, American Chemical Society.

Wong et al. reported that the electrochemical etching via applying a positive voltage can form S vacancies in the multilayer 2H-MoS_2_ nanosheets, which act as electron donors to promote the 2H/1T phase transition [59]. As shown in the Raman spectra (Figure 12a), the pristine 2H-MoS_2_ presents four main characteristic peaks at 282, 380, 406, and 450 cm^−1^ that correspond to the *E*_1g_, $E_{2g}^{1}$, and *A*_1g_ vibration modes and the longitudinal acoustic phonon mode of 2H-MoS_2_, respectively. Once the positive potential is applied to treat the original 2H-MoS_2_, several new vibration modes can be observed at 151, 221, and 326 cm^−1^, which are attributed to the J1, J2, and J3 peaks of the 1T phase. In addition, with the improvement in the applied potential, the above three characteristic peaks become more and more obvious, indicating the increase in 1T phase content. However, it is worth noting that for the S1.6-160 and S2.2-160 samples, there appear high-frequency vibration modes at 794, 847, and 880 cm^−1^, which are related to aqueous MoO_3_. The strength of MoO_3_ for the S2.2-160 is higher than that for the S1.6-160, confirming the increased O doping level in the S2.2-160 sample. Besides, the authors further combined the theoretical calculation to analyze the electronic energy band structure of all MoS_2_ samples (Figure 12b). The band gap of 2H-MoS_2_ with the 1T phase and O doping is 0.80 eV, which is narrower than those of pristine 2H-MoS_2_ (1.90 eV), pure O-doped 2H-MoS_2_ (1.62 eV), and 2H-MoS_2_ with the 1T phase (0.99 eV) (Figure 12c,d). Therefore, the synergistic effect of the 1T phase and O doping can improve the electrical conductivity of MoS_2_ nanosheets, eventually leading to the significantly reduced overpotential and Tafel slope for HER electrocatalysis (Figure 12e,f).

#### 3.3.2. Synergistic Effect of Phase Transition and S Vacancies Induced by Doping

**Figure 13:**
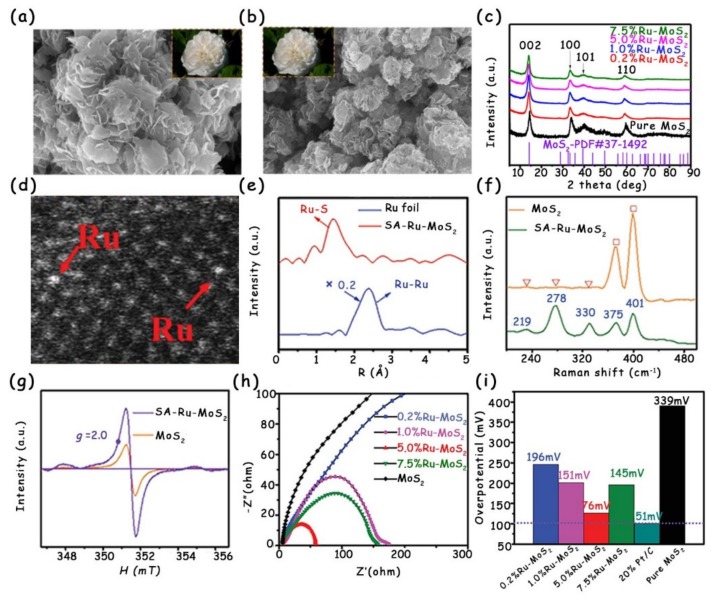
SEM images of (**a**) single-atom Ru-doped (SA-Ru)-MoS_2_ and (**b**) pure MoS_2_ nanosheets. Scale bar: 200 nm. (**c**) XRD patterns of pure MoS_2_ and Ru-MoS_2_ catalysts with different Ru contents. (**d**) HAADF-STEM image of SA-Ru-MoS_2_ showing the single Ru atoms. (**e**) k^2^-Weighted EXAFS spectra of SA-Ru-MoS_2_ and Ru foil. (**f**) Raman spectra and (**g**) electron spin resonances of SA-Ru-MoS_2_ and pure MoS_2_. (**h**) Electrochemical impedance spectroscopy (EIS) of pure MoS_2_ and SA-Ru-MoS_2_ with different Ru doping concentrations. (**i**) Overpotentials at 10 mA cm^−2^ of pure MoS_2_ and SA-Ru-MoS_2_ with different Ru contents. Reproduced with permission from Ref. 33. Copyright © 2018, Wiley-VCH Verlag GmbH & Co. KGaA, Weinheim.

Doping can induce the partial phase transition and generation of S vacancies, which adjust the local electronic structure of MoS_2_ and form the new interstitial states, thus improving the electronic conductivity [33]. Recently, Cao’s group successfully modified MoS_2_ nanosheets via single-atom Ru doping using a simple one-step impregnation method [33]. The SEM images (Figure 13a,b) and XRD patterns (Figure 13c) show that both morphology and crystal structure after Ru doping are similar to those of original MoS_2_, indicating that the modified strategy does not cause excessive damage to MoS_2_. The authors further combined the high-angle annular dark-field scanning transmission electron microscopy (HAADF-STEM) image (Figure 13d) and Fourier-transformed R-space spectrum (Figure 13e) to analyze the existence form of Ru in MoS_2_. All the above characterization results together prove that Ru is single-atomically anchored in the MoS_2_ plane by replacing Mo sites and coordinating with S atoms. Interestingly, in comparison with the original MoS_2_ consisting of the pure 2H phase, there contain both 2H and 1T phases in the single-atom Ru-doped MoS_2_ (SA-Ru-MoS_2_), as presented in the Raman spectra (Figure 13f). In addition, the paramagnetic signal detection of electron spin resonance confirms that abundant S vacancies exist in the SA-Ru-MoS_2_ (Figure 13g). Due to the co-existence of the 1T phase and S vacancies induced by Ru doping, all the SA-Ru-MoS_2_ with different Ru doping concentrations exhibit much lower charge transfer resistance (R_ct_) values than those of the original MoS_2_ (Figure 13h). Especially for the SA-Ru-MoS_2_ sample with 5.0% Ru doping, it possesses the best charge transfer property. To deeply understand the effect of single-atom Ru doping on the conductivity of SA-Ru-MoS_2_, the authors also performed electronic structure analysis. The S vacancies and single-atom Ru doping together generate the interstitial states near the Fermi level, which result in increased electrical conductivity. Ultimately, the 5.0%-Ru-MoS_2_ only requires a low overpotential of 76 mV to reach the HER current density of 10 mA cm^−2^ (Figure 13i).

**Figure 14:**
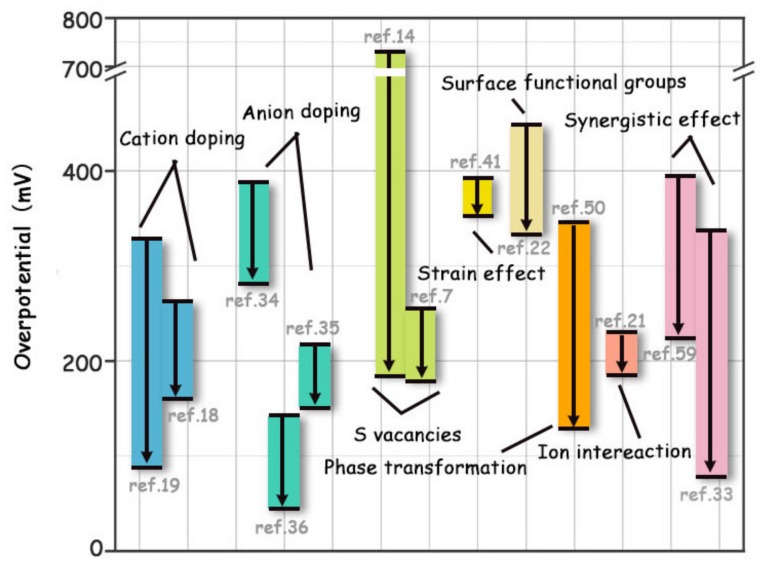
Overpotentials to drive the HER current density of 10 mA cm^−2^ of MoS_2_ before and after various modification strategies.

To visually compare the effect of various modification strategies on the HER activity enhancement of original MoS_2_, the overpotentials at 10 mA cm^−2^ before and after modification are given in Figure 14.

## 4. Summary and Outlook

This review has summarized the recent modification strategies for boosting the HER performance of original MoS_2_, mainly including the following three aspects: (1) Improving the intrinsic activity of catalytic sites by optimizing the electronic structure of the edge, or activating the inert basal plane to improve the hydrogen adsorption behavior; (2) increasing the number of active sites through introducing cracks or holes into the MoS_2_ layers; (3) enhancing the electrical conductivity by forming new electronic states near the Fermi level and, thus, narrowing the bandgap of MoS_2_. Although these strategies have effectively modified the pristine MoS_2_ and made their HER activity close to that of commercial Pt-based catalysts, there are still some issues to be solved before using these modified MoS_2_ in industry. The first is the electrocatalytic stability of materials. Practical industrial applications require catalysts with excellent long-term stability, not limited to several tens of hours in the laboratory, and strong corrosion resistance. The second challenge is the large-scale preparation. Due to the strict and cumbersome modification reaction conditions, and limited reactor size of some strategies, it may not be easy to prepare the modified MoS_2_ catalysts on a large scale. Lastly, the further improvement in HER performance of the original MoS_2_ remains challenging. Despite the modified MoS_2_ catalysts exhibiting obviously improved HER activity, it is usually difficult to surpass that of the noble metal Pt, especially in alkaline media.
